# Whole-Genome Sequence of the Fish Virulent Strain *Streptococcus iniae* IUSA-1, Isolated from Gilthead Sea Bream (*Sparus aurata*) and Red Porgy (*Pagrus pagrus*)

**DOI:** 10.1128/genomeA.00025-13

**Published:** 2013-03-14

**Authors:** Fatima El Aamri, Félix Acosta, Fernando Real, Daniel Padilla

**Affiliations:** Institute of Animal Health (IUSA), University of Las Palmas de Gran Canaria, Arucas, Arucas, Spain

## Abstract

*Streptococcus iniae* is a major fish pathogen that produces invasive infections that result in economic losses in aquaculture. In this study, the draft genome sequence of *Streptococcus iniae* strain IUSA-1, isolated from a natural outbreak affecting gilthead sea bream (*Sparus aurata*) and red porgy (*Pagrus pagrus*), is presented.

## GENOME ANNOUNCEMENT

*Streptococcus iniae* is an important Gram-positive bacterium that is associated with acute and chronic mortality in marine and continental aquaculture (1), affecting more than 30 fish species (2, 3, 4), and has been reported in Asia, Australia, America, and Europe (3, 4). *S. iniae* causes invasive infections, producing high economic losses in aquaculture. *S. iniae* is also a zoonotic pathogen, causing soft tissue infections and sepsis in humans (5, 6). Gilthead sea bream and red porgy affected by *S. iniae* IUSA-1 showed clinical signs of hemorrhagic septicemia, lethargy, anorexia, abnormal swimming, exophthalmia, and sudden death, with mortality rates over 25% in red porgy and 10% in gilthead sea bream, and with acute meningoencephalitis as the main lesion. The strain was grown statically at 25°C in Trypticase soy agar supplemented with 0.5% (vol/vol) yeast extract. Cells were grown until the exponential phase for 24 h and harvested for the purification of genomic DNA using a DNeasy blood and tissue kit (Qiagen, Hilden, Germany). A whole-genome shotgun strategy was used with a Roche 454 GS-FLX Titanium sequencing platform (Macrogen, South Korea). A total of 137,532 reads (56,754,481 nucleotides) were generated, reaching a depth of 27.3-fold genome coverage. By using Roche’s software GS *de novo* assembler (v2.6), they were assembled into 455 contigs; 296 of these were >500 bp, with bases having quality scores of ≥40. The genome of *S. iniae* IUSA-1 is composed of 2.22 Mb, with an average G+C content of 36.3%.

### Nucleotide sequence accession numbers.

This Whole Genome Shotgun project has been deposited at DDBJ/EMBL/GenBank under the accession no. AOCT00000000. The version described in this paper is the second version, AOCT02000000.

## References

[1] Bercovier H Ghittino C Eldar A 1997 Immunization with bacterial antigens: infections with streptococci and related organisms, p 153–160 *In* Gudding L Midtlyng B, Fish vaccinology. Developments in Biologicals, vol 90. Karger, Basel, Switzerland 9270844

[2] Klesius P Evans J Shoemaker C Yeh H Goodwin AE Adams A Thompson K 2006 Rapid detection and identification of *Streptococcus iniae* using a monoclonal antibody-based indirect fluorescent antibody technique. Aquaculture 258:180–186

[3] Agnew W Barnes AC 2007 Streptococcus iniae: an aquatic pathogen of global veterinary significance and a challenging candidate for reliable vaccination. Vet. Microbiol. 122:1–15 17418985 10.1016/j.vetmic.2007.03.002

[4] El Aamri F Padilla D Acosta F Caballero M Roo J Bravo J Vivas J Real F 2010 First report of *Streptococcus iniae* in red porgy (*Pagrus pagrus*, L). J. Fish Dis. 33:901–905 21504081 10.1111/j.1365-2761.2010.01191.x

[5] Weinstein MR Litt M Kertesz DA Wyper P Rose D Coulter M Facklam R Ostach C Willey BM Borczyk A Low DE 1997 Invasive infections due to a fish pathogen, *Streptococcus iniae*. *S. iniae* study group. N. Engl. J. Med. 337:589–594 9271480 10.1056/NEJM199708283370902

[6] Lau SK Woo PC Luk WK Fung AM Hui WT Fong AH Chow CW Wong SS Yuen KY 2006 Clinical isolates of *Streptococcus iniae* from Asia are more mucoid and β-hemolytic than those from North America. Diagn. Microbiol. Infect. Dis. 54:177–181 16427243 10.1016/j.diagmicrobio.2005.09.012

